# Protective Role of Melatonin in Neonatal Diseases

**DOI:** 10.1155/2013/980374

**Published:** 2013-11-19

**Authors:** Eloisa Gitto, Lucia Marseglia, Sara Manti, Gabriella D'Angelo, Ignazio Barberi, Carmelo Salpietro, Russel J. Reiter

**Affiliations:** ^1^Neonatal Intensive Care Unit, Department of Pediatrics, University of Messina, Via Consolare Valeria, 98125 Messina, Italy; ^2^Unit of Pediatric Genetics and Immunology, Department of Pediatrics, University of Messina, Via Consolare Valeria 1, 98125 Messina, Italy; ^3^Department of Cellular and Structural Biology, University of Texas Health Science Center at San Antonio, San Antonio, TX 40729, USA

## Abstract

Oxidative stress contributes to the severity of several newborn conditions to the extent that Saugstad coined the phrase “oxygen radical diseases of neonatology.” In order to counteract free radicals damage many strategies to augment antioxidant status in ill-term and preterm infants have been proposed and several medications have been experimented with mixed results. Several studies have tested the efficacy of melatonin to counteract oxidative damage in diseases of newborns such as chronic lung disease, perinatal brain injury, necrotizing enterocolitis, and retinopathy of prematurity, giving promising results. The peculiar perinatal susceptibility to oxidative stress indicates that prophylactic use of antioxidants as melatonin could help to prevent or at least reduce oxidative stress related diseases in newborns. However, more studies are needed to confirm these beneficial effects.

## 1. Introduction 

Oxygen- and nitrogen-derived metabolites, collectively termed reactive oxygen species (ROS) and reactive nitrogen species (RNS), are persistently produced in aerobic organisms. When generated in excess, ROS/RNS mutilate molecules and are important mediators of cell and tissue damage [1, 2]. The resulting damage, which is unavoidable, is referred to as oxidative stress caused by free radicals. Free radicals are highly unstable and, normally, their formation is controlled by several beneficial compounds known as antioxidants; these protective molecules are part of the antioxidative defence system. There is a critical balance between free radical generation and antioxidant defences. Free radicals production is the endpoint of a cascade of several biochemical events such as hypoxia, hyperoxia, ischemia, and inflammation. A direct relation has been demonstrated between the degree of hypoxia and the severity of oxidative stress due to free radicals production during hypoxia in fetal life [3]. Free radical reactions lead to the oxidation of lipids, proteins, and polysaccharides and to DNA damage (fragmentation, base modifications, and strand breaks); as a consequence, radicals have a wide range of biologically toxic effects. Oxidative stress can be defined as an imbalance between the amount of ROS and the intracellular and extracellular antioxidant protection systems. Newborns, especially if born prematurely, are very susceptible to free radical oxidative damage, for many several reasons [4]. In fact (a) infants at birth are naturally exposed to the hyperoxic challenge due to the transition from the hypoxic intrauterine environment to extrauterine life, and this gap is even more significant for newborns that require supplemental oxygen during resuscitation in the delivery room; (b) they are more susceptible to infection, especially if born prematurely; (c) they have reduced antioxidant defence processes; (d) they possess high levels of free iron that enhances the Fenton reaction causing the production of highly toxic radicals [5]. Oxidative stress likely contributes to the severity of several newborn conditions to the extent that Saugstad [6, 7] coined the phrase “oxygen radical diseases of neonatology.” The idea implies that oxidative stress affects a variety of organs, often simultaneously, and gives rise to different signs according to the organ most damaged. He includes bronchopulmonary dysplasia/chronic lung disease (CLD), retinopathy of prematurity (ROP), and necrotizing enterocolitis (NEC) in this category. Subsequently, it became clear that free radicals are involved in periventricular leukomalacia [8] as well as in influencing the ductus arteriosus and pulmonary circulation [9–11]. If the concept of “oxygen radical diseases of neonatology” has validity, it meant that the above-mentioned conditions are *not *different disease entities but are simply different *organ *manifestations of the same complex processes of oxidative stress and metabolism. In order to counteract free radicals damage many strategies to augment antioxidant status in ill-term and preterm infants have been proposed and several medications have been experimented with mixed results. Melatonin is an endogenously produced indolamine principally synthesized in the pineal gland from the neurotransmitter serotonin [12]. It has recently been recognised as a “ubiquitously distributed and functionally diverse molecule” [13]. In fact, melatonin plays a key role in a variety of important physiological functions, including regulation of circadian rhythms, as well as visual, reproductive, cerebrovascular, neuroendocrine, and neuroimmunological actions [14]. Furthermore, melatonin is a highly effective antioxidant and free-radical scavenger [12]. It is by now well known that melatonin directly scavenges ROS detoxifying against the highly reactive hydroxyl radicals. Melatonin and its metabolites efficiently interact with various ROS/RNS, as well as with organic radicals, upregulate antioxidant enzymes (including glutathione peroxidases and glutathione reductase), and downregulate prooxidant enzymes (nitric oxide synthases and lipoxygenases) [15]. Thus, melatonin prevents lipid peroxidation (LPO), reduces mitochondrial hydroperoxide levels, and restores glutathione homeostasis (GSH) [16]. Furthermore, melatonin reduces nuclear factor kappa-light-chain enhancer of activated B cells binding to DNA, probably by preventing its translocation to the nucleus [17]. Finally, melatonin reduces the production of proinflammatory cytokines and chemokines and has been shown to reduce recruitment of polymorphonuclear leukocytes to inflammatory sites [18]. High endogenous melatonin levels have been observed in critically ill children in comparison to normal age-matched subjects and this finding has been considered as a response to counteract the elevated oxidative stress associated with serious diseases [19]. In light of these properties, melatonin has been used as an adjuvant in the treatment of free radical disease in term and preterm newborns. Moreover, since preterm infants are melatonin deficient, administration of the compound may provide the necessary levels to assure their health and well-being [20].

The pharmacokinetic profile of melatonin in preterm infants differs from that of adults; therefore dosage of melatonin for preterm infants cannot be extrapolated from adult studies. Recently, Merchant et al. demonstrated that the half-life of melatonin in the preterm population is approximately 15 h. In fact, melatonin concentrations closest to adult concentrations were achieved with a 2 h infusion of 0.1 mg kg^−1^ h^−1^ of melatonin [21].

## 2. Melatonin and Perinatal Brain Injury 

Injury to the fetal brain is a major contributor to morbidity and mortality in preterm and term [22] infants. Neonatal haemorrhagic brain injury (such as intraventricular haemorrhage) [23] and white matter brain injury (such as periventricular leukomalacia) are often causes of long term neurosensory disabilities, including cerebral palsy. The pathogenesis of brain injury is known to be complex and multifactorial, with a number of interrelated pathways contributing to central nervous system cellular dysfunction and, in this contest, the free radical induced damage appears to have a crucial role [24]. Neuropathological studies indicate that many critical neuronal groups are more vulnerable to hypoxic ischemic injury in newborns (immature brain) than in adults, particularly related to enhanced density and function of excitatory aminoacid receptors as well as enhanced vulnerability to be attacked by ROS and RNS [25]. The immature brain has more blood vessels, higher water content, lower myelin, a poorly developed cortex, and a more prominent germinal matrix than the mature brain [26]. The degree of damage depends on the region of the brain that is affected, the severity of the insult, and the stage of development. The morbidity and mortality of infants, especially if preterm, are strongly affected by their ability to maintain physiologic homeostasis and to counteract the effects of free radicals [27]. During the last decade, melatonin has started to be considered as an attractive option in order to minimize as much as possible the neurological sequelae from hypoxic-ischemic brain injury. Melatonin appears as very interesting drug, because of its ability to cross all physiological barriers reaching subcellular compartments and its efficacy and safety profile [28–31]. The neuroprotective effects of melatonin in the fetal brain have been assessed in many animal models. Following intrauterine asphyxia (via umbilical cord occlusion), melatonin administration to both preterm and near-term fetal sheep has been shown to reduce oxidative stress [32] and attenuate cell death (including apoptosis) in the fetal brain, in association with a reduced inflammatory response [33]. Systemic administration of melatonin, following acute neonatal haemorrhagic brain injury in rats, has additionally been shown to protect against posthemorrhagic consequences of brain atrophy [23]. Importantly, melatonin has been shown to improve functional outcomes following such brain injury-ameliorating cognitive and sensorimotor dysfunction in the juvenile rat [23]. Husson et al. demonstrated that melatonin, acting on its receptors and through adenylate cyclase inhibition, was neuroprotective in a newborn mouse model of excitotoxic white matter lesions mimicking human PVL. In fact, mice that received intraperitoneal melatonin had an 82% reduction in size of ibotenate-induced white matter cysts when compared with controls. Moreover, even if melatonin did not prevent the initial appearance of white matter lesions, it did promote secondary lesion repair. Axonal markers supported the hypothesis that melatonin induced axonal regrowth or sprouting [34].

Fulia et al., in the first study where melatonin was given to human newborns, measured a product of lipid peroxidation, malondialdehyde, and the nitrite/nitrate levels in the serum of asphyxiated newborns before and after treatment with the antioxidant melatonin given within the first 6 hours of life. Following treatment of asphyxiated newborns with melatonin, there was a significant reduction in the products of lipid peroxidation at both 12 and 24 hours after treatment. Nitrite/nitrate levels dropped significantly in treated infants, while they remained high and even further increased in the asphyxiated infants not given melatonin. The protective actions of melatonin in this study may relate to its antioxidant properties as well as to the ability of melatonin to increase the efficiency of mitochondrial electron transport [35]. Nowadays, hypothermia is recognized as an efficacious treatment modality for neonatal hypoxic ischemic encephalopathy. The use of synergic strategies, such as the association between hypothermia and melatonin supplementation, may lead to a larger neuroprotective effect on the brain, thus improving the neonatal outcome. In this regard, Robertson et al. have recently shown that melatonin administration to newborn piglets augments hypothermic neuroprotection by improving cerebral energy metabolism and by reducing brain damage [36]. In this study 5 mg/kg/h melatonin is administered intravenously 10 min after the end of transient hypoxia ischaemia over 6 h and is repeated at 24 h augmented hypothermic neuroprotection based on improved cerebral energy metabolism using magnetic resonance spectroscopy biomarkers (deep grey matter lactate/N-acetyl aspartate and lactate/creatine). Melatonin-augmented hypothermia was also founded to increase levels of cerebral ATP. The discovery that melatonin also crosses the placenta is a prerequisite to hypothesize a neuroprotective role of melatonin in fetus at risk for hypoxic ischemic injury [36]. The maternal-fetal transfer of melatonin in humans has been verified by measuring the concentration of melatonin in the fetal circulation after its administration to near-term pregnant women. The oral administration of 3 mg of melatonin led to marked increases in the serum levels with maximum values being observed 2 h after drug administration; serum levels of melatonin in the umbilical vein were closely correlated with those in the maternal vein [37]. These findings support the idea that melatonin, easily transferred from the maternal to the fetal circulation, could be harmful to prevent the free radical damage in fetus at risk of asphyxia.

## 3. Melatonin and Chronic Lung Disease 

Lung injury in the neonatal period has multiple etiologic factors—genetic, hemodynamic, metabolic, nutritional, mechanical, and infectious mechanisms—which act in a cumulative and synergic way. Free radical generation is largely recognized as the major cause of lung damage. Oxidative stress is the final common endpoint for a complex convergence of events, some genetically determined and some triggered by in utero stressors. Inflammatory placental disorders and chorioamnionitis also play an important role due to the coexistence of inflammatory and oxidative lesions. In addition, the contribution of airway inflammation has been extensively studied [38].

Although oxygen therapy is essential in the treatment of respiratory disorders of newborn, hyperoxic exposure itself induces excessive production of ROS/RNS in the respiratory system. The exposure of immature lungs to prolonged periods of high levels of inspired oxygen is accepted as an important contributor to the development of CLD through both free radical effects on endothelial and epithelial cell barriers that induce pulmonary oedema and trigger mechanisms that lead to activation and accumulation of inflammatory cells [39]. In these situations, it is believed that melatonin could act as antioxidant limiting the radical induced damage. In fact, recently, it has been demonstrated that melatonin prevented the hyperoxia-induced increases of the myeloperoxidase (MPO), malondialdehyde, nitrite/nitrate, levels and attenuated the hyperoxia-induced depletion of antioxidant enzyme activities in the damaged lung tissue of rats [40]. In this study, nocturnal administration of 4 mg/kg melatonin seemed to prevent CLD associated histopathological alterations such as reduced total number of alveoli and interstitial fibrosis as assessed by semiquantitative morphological indices of radical alveolar counts and collagen fiber staining [40]. However, the fact that premature infants develop CLD without being exposed to high concentrations of supplemental oxygen raises the question as to whether hyperoxic insult contributes to the development of CLD. It is well known that pulmonary damage depends in large part on the ventilatory strategies used. Ventilator-associated lung injury is believed to result from the use of high pressure, thus, the term barotraumas. This trauma is noted to involve free-radical damage [39].

Gitto et al. [41] tested whether newborns with RDS differently ventilated and receiving melatonin had lower proinflammatory cytokines in tracheobronchial aspirate and better clinical outcome in terms of developing CLD. In particular, it was demonstrated that newborns mechanically ventilated in Pressure Support Ventilation mode with Guarantee Volume and receiving melatonin presented a greater reduction of serum levels of inflammatory cytokines than did newborns ventilated in conventional mode or in oscillatory ventilation receiving melatonin or diluent alone. The measured inflammatory cytokines were most markedly elevated in infants mechanically ventilated but not given melatonin. Interleukins 6, 8 and tumor necrosis factor *α* were significantly higher in infants who developed CLD. Thus the antioxidant and anti-inflammatory effects of melatonin could be effective in the prevention of CLD in ventilated newborns. Since melatonin has a protective effect against ROS [16] and RNS [42], it could reduce the ROS and RNS production or ROS and RNS-induced progression in the early inflammatory phase. Accumulation of inflammatory cell produces significant damage in CLD and antioxidant agents may protect against tissue damage by reducing neutrophils influx into the tissue [43]. The inhibitory effects of melatonin on the accumulation of leukocytes into the lung manifested by the reduction in MPO activity in lung tissue may contribute to a further protection of the lung from free radical damage produced by leukocytes. The upregulation of melatonin receptor mRNA in lipopolysaccharide induced lung injury [44] suggests that ligand-receptor binding reaction might be involved in the mechanism through which melatonin prevents lung injury. In this regard, melatonin is promising for the treatment of preterm infants with lung disease.

## 4. Melatonin and Necrotizing Enterocolitis (NEC)

Necrotizing enterocolitis (NEC) is primarily a disease process of the gastrointestinal tract of premature neonates that results in inflammation and bacterial invasion of the bowel wall. The prevalence of the disorder is about 7% among infants with a birth weight between 500 and 1500 g. The estimated rate of death is between 20 and 30% [45]. Several etiologic factors have been identified in the pathogenesis of NEC: immaturity, hypoxia/ischemia, hyperosmolar feedings, and bacterial colonization, as well as oxidative stress [46]. Mediators that may be involved in the pathogenesis of NEC including platelet activating factor (PAF), intestinal toll-like receptors, tumor necrosis factor *α*, interleukins 1*β*, 6, 8, 10, and 12, lipopolysaccharide, nitric oxide (NO), RNS, and oxygen derived free radicals that amplify the inflammatory response via several mechanisms including peroxidation of membrane lipids as well as oxidation of proteins and DNA. Recent studies, using animal model, show that there is a clear correlation between NEC and oxidative stress [47, 48]. Perrone et al., measuring in cord blood nonprotein bound iron (NPBI), advanced oxidation protein products, and total hydroperoxides, showed that the determination of oxidative-stress biomarkers can be useful in identifying babies at high risk for NEC [49]. Therefore to reduce or prevent the incidence and/or severity of NEC may be useful include use of inflammatory mediator antagonists and antioxidants such as melatonin.

Melatonin is also produced in a variety of tissues, including the intestines, especially after feeding, mainly in serotonin-rich enteroendocrine cells (enterochromaffin cells) [50]. The beneficial effects of melatonin in preventing gastrointestinal disturbances were studied in mice [47, 48]. The pathophysiology of NEC involves a complex interaction of proinflammatory cytokines [50]. Studies demonstrate that tumor necrosis factor *α* and interleukin 1*β* were reduced in animal model affected NEC and treated with melatonin [47]. Melatonin reversed lipopolysaccharide induced motility disturbances. Therefore melatonin normalized the altered lipid peroxidation, ROS/RNS, p38 mitogen-activated protein kinase activation, iNOS transcription and expression, and nitrite production in intestinal tissue from mice [48]. The major mechanism of melatonin on cytoprotection effect includes free radical scavenging activity and activation of cyclooxygenase-prostaglandin enzyme system, exercising cytoprotective and inflammation activities on gastrointestinal system mucosa. On NEC model in newborn rats, it has used the combination of melatonin and prostaglandin. This association may be useful to save lives [50]. The final pathway in NEC pathogenesis involves free radical injury. Based on these observations, melatonin should be considered as a potentially safe approach to NEC treatment in infants. 

## 5. Melatonin and Retinopathy of Prematurity

Oxygen-induced oxidative stress has damaging effects in the perinatal period. This evidence occurs in babies with retinopathy of prematurity (ROP) too [51]. ROP is a proliferative disease of the retinal vasculature in premature infants that may cause severe visual loss, and it is a major cause of blindness in newborns. Incidence of ROP is more common in premature infants exposed to high concentration of oxygen as it causes generation of free radicals. Following the intuition of Ashton and Cook in 1954, several many studies established the relationship between a high-oxygen saturation and halting retinal blood vessel development [52, 53]. On the other hand, hypoxia as well is known to play a critical role in the genesis of retinopathy of prematurity [54]. In 2013, Kaur et al. [55] first investigated, in neonatal rat, the effect of melatonin treatment in the prevention of the retinal ganglion cell (RGC) death in the developing retina following a hypoxic insult. They founded that cultures of hypoxic microglial cells treated with melatonin showed a significant reduction in the release of inflammatory cytokines as compared to untreated hypoxic cells. They also observed an increased release of cytochrome c into the cytosol of the hypoxic retina followed by activation of cytosolic caspases that induced apoptosis. Cytochrome c, caspase-3 levels, and apoptosis in the RGCs of hypoxic retinas were significantly reduced after melatonin administration. The study also showed that lipid peroxidation (LPO) was increased in the hypoxic retina, while conversely, GSH levels were decreased, confirming again that melatonin is a potent antioxidant protecting tissues and cells against damage from oxidative stress [55]. However, several studies showed that melatonin production is minimal in newborns and its production gradually wanes in aging individuals. These patients are possibly at risk of contracting degenerative eye diseases that are free radical based. Supplementation with melatonin, a potent antioxidant, preserves their visual functions [56]. In fact, by activating different melatonin receptor subtypes, melatonin modulates activities of retinal neurons. Different subtypes of melatonin receptors are present on major types of retinal neurons, and the expression of these receptors is highly species and neuron subtype dependent [57]. The neuroprotective effect of melatonin is mediated by the inhibition of contributing factors to hypoxia such as Hypoxia Inducible Factor-1*α* (HIF-1*α*) too [58].

Although further studies are needed to confirm the utility of supplementation of endogenous antioxidants as melatonin in damaged retinal tissue, these results may have therapeutic implications for the management of retinopathy of prematurity.

## 6. Conclusion

As Saugstad intuited, free radicals damage has a well-known role in the pathogenesis of several diseases of term and preterm newborns. Accordingly the preventive and therapeutic strategies have focused on avoiding the reactive oxygen species as well as using antioxidants. The peculiar perinatal susceptibility to oxidative stress indicates that prophylactic use of antioxidants as melatonin could help to prevent or at least reduce oxidative stress related diseases in newborns. Several studies have tested the efficacy of melatonin to counteract oxidative damage in “oxygen radical diseases of newborn” such as CLD, perinatal brain injury, NEC, and ROP, giving promising results. However, more studies are needed to confirm the beneficial effects of melatonin in the oxidative stress in perinatal period. 
